# Supplementary material for: SLX4IP and telomere dynamics dictate breast cancer metastasis and therapeutic responsiveness
Source: Life Sci Alliance. 2020 Feb 18;3(4):e201900427. doi: 10.26508/lsa.201900427 (PMC7032570; doi:10.26508/lsa.201900427)
Supplement: Supplementary file 1 [file LSA-2019-00427_TableS1.docx]

**Supplementary Table S1. Sequences of shRNAs and CRISPR constructs.**

| **Target Gene** | **Target Sequence** |
| --- | --- |
| Mouse SLX4IP shRNA1 | 5’-CCGGTCCATGGAGTGTCTGATTATTCTCGAGAATAATCAGA CACTCCATGGATTTTTG |
| Mouse SLX4IP shRNA2 | 5’-CCGGTCACCGTCTCCTGGTACAAAGCTCGAGCTTTGTACCAG GAGACGGTGATTTTTG |
| Mouse scrambled shRNA | 5’-CCTAAGGTTAAGTCGCCCTCGCTCGAGCGAGGGCGACTTAA CCTTAGG |
| Mouse TERT CRISPR 1 | 5’-CCGCTGGCAACCTTTGTGCGGCG |
| Mouse TERT CRISPR 2 | 5’-CCGCACTTTGGTTGCCCAATGCC |
| Human SLX4IP CRISPR | 5’-TTCCTCCCAGTGCAAAGCTC |
