# Supplementary material for: SLX4IP and telomere dynamics dictate breast cancer metastasis and therapeutic responsiveness
Source: Life Sci Alliance. 2020 Feb 18;3(4):e201900427. doi: 10.26508/lsa.201900427 (PMC7032570; doi:10.26508/lsa.201900427)
Supplement: Supplementary file 2 [file LSA-2019-00427_TableS2.docx]

**Supplementary Table S2. Primer sequences.**

| **Target** | **Forward Sequence** | **Reverse Sequence** |
| --- | --- | --- |
| VBIM Gene ID (PCR) | 1: 5’-CAACCCACAAGGAGACGAC CT  2: 5’-CGTTCGTCCTCACTCTCTT CC  3: 5’-GTCTGCGAGGGCCAGCTGG AT | 1: 5’-GCCCAGGTCCAATCACGCN NNNNN  2/3: 5’-GCCCAGGTCCAATCACGC |
| Mouse SLX4IP (qRT-PCR) | 5’-AAACAGCGCAAGCCATCAAG | 5’-GCAAGCTGACTAACACACACC |
| Mouse GAPDH (qRT-PCR) | 5’-CAACTTTGGCATTGTGGAAGG GCTC | 5’-GCAGGGATGATGTTCTGG GCAGC |
| Mouse TERT (qRT-PCR) | 5’-ACAAGCTCCTGTCGGTCTTG | 5’-AACACGCTGGTCAAAGGGAA |
| Mouse TR (RIP) | 5’-CGCTGTTTTTCTCGCTGACTT | 5’-CTCCTGCGCTGACGTTTGTT |
| Telomere C-circles (CCA) | 5’-CGGTTTG(TTTGGG)_5_TT | 5’-GGCTTGCC(TTACCC)_5_T |
| Mouse 36B4 (CCA) | 5’-ACTAATCCCGCCAAAGCAACC | 5’-GTAGCGGTTTTGCTTTTTCA TCCT |
| Human 36B4 (CCA) | 5’-CAGCAAGTGGGAAGGTGTAA TCC | 5’-CCATTCTATCATCAACGGG TACAA |
| Mouse ATRX (qRT-PCR) | 5’-TCCAGCACTATCTGCGGTGA | 5’-ACTTGTTTCCACTCATGGGCT |
| Mouse Daxx (qRT-PCR) | 5’-CCCCTCTGACCTTACAAACAC | 5’-GGGCCTCCTGCAAATACGAG |
| Mouse ATRX (ChIP) | 5’-CTGCAACCCTGACATCTAAGC | 5’-TCCAAATCGTTTCCCCCTCAT |
| Mouse Daxx (ChIP) | 5’-TTCACAGGCACTTGCACTCA | 5’-CAATGGCTGACCGGACTCTT |
| Mouse GAPDH (ChIP) | 5’-CCCTTCCCACCCTGTTCATC | 5’-GGCTGCACCCCAAATCTAGT |
| Mouse MyoD (ChIP) | 5’-GCACTGCCACCGATTCATTT | 5’-GAGACTCAAGGCTACGGGAC |
| Mouse GLB1 (qRT-PCR) | 5’-ACCCAGAGGACATTTAAGCT CG | 5’-TAGCATTCAGCCCAGCCATC |
| Mouse TERT TSS (ChIP) | 5’-ACTTTGGTTGCCCAATGC | 5’-AAGGAAAGGTCGGCAGGT |
| Mouse TERT TSS-1000 (ChIP) | 5’-CCCTATTTCCCAGAGATTCAAA | 5’-GGGGCATATGTAATGACACGA |
| Mouse TERT TSS+1000 (ChIP) | 5’-CAGGAACTGATGTGGAAGAT GA | 5’-AGACCAGCCATGCTCACC |
| Human SLX4IP (qRT-PCR) | 5’-AGGTACTACTCCGCCAGTGA | 5’-TATGATGGTGGCGGGGTTTG |
| Human TERT (qRT-PCR) | 5’-GAGAACAAGCTGTTTGCGGG | 5’-AAGTTCACCACGCAGCCATA |
| Human GAPDH (qRT-PCR) | 5’-TCCATGACAACTTTGGTATTC GT | 5’-AGTAGAGGCAGGGATGATGTT |
| SLX4IP asRNA (PCR) | 5’-GTTTAAACGGCCAGCTGGAT GGTGGG | 5’-GTTTAAACGGACGCTCTGAAG GAAATTGTT |
| SLX4IP asRNA (qRT-PCR) | 5’-TTTGCCTGGTTGAGCCTTCA | 5’-TCAAACAGAATGTGTAGCTTT CC |
| Mouse SLX4IP cDNA (PCR) | 5’-GTCGACATGGCATCTAAGAAA TTTGCCG | 5’-GCGGATATCGTTTTTCTTGCC AC |

CCA, C-circle assay
